# microRNA‐637 promotes apoptosis and suppresses proliferation and autophagy in multiple myeloma cell lines via NUPR1

**DOI:** 10.1002/2211-5463.13063

**Published:** 2020-12-30

**Authors:** Xuanxin Chen, Anmao Li, Qian Zhan, Zizi Jing, Yiyu Chen, Jianbin Chen

**Affiliations:** ^1^ Department of Hematology the First Affiliated Hospital of Chongqing Medical University China; ^2^ The Center for Clinical Molecular Medical Detection the First Affiliated Hospital of Chongqing Medical University China; ^3^ Institute of Life Sciences Chongqing Medical University China

**Keywords:** apoptosis, autophagy, microRNA‐637 (miR‐637), multiple myeloma, NUPR1, proliferation

## Abstract

Multiple myeloma (MM) is a heterogeneous disease with poor prognosis. Increasing evidence has revealed that microRNAs (miRNAs) are strongly associated with the pathogenesis and progression of MM. Here, we investigated the role of microRNA‐637 (miR‐637) in MM to identify potential therapeutic targets. We measured the expression of miR‐637 in bone marrow samples of MM patients and MM cell lines by quantitative real‐time PCR and western blot. The effect of miR‐637 on proliferation and apoptosis of MM primary cells was also investigated. Analyses of four bioinformatics databases showed that miR‐637 is associated with nuclear protein 1 (NUPR1) in MM cells, which was confirmed by luciferase reporter assay. We found that the overexpression of miR‐637 suppressed the development of MM. miR‐637 mimics increased the levels of Bax, cleaved caspase 3, and P62, and decreased the levels of Bcl2 and LC3. Additionally, luciferase reporter assays were performed to demonstrate that NUPR1 is the main target of miR‐637 in MM cells. Overexpression of NUPR1 reversed the effects of miR‐637 mimics in MM cells. Our results suggest that miR‐637 inhibits cell proliferation and autophagy, and promotes apoptosis in MM cells by targeting NUPR1. Our findings also suggest that miR‐637 may have potential as a novel molecular therapeutic target for MM treatment.

## Graphical abstract

Multiple myeloma (MM) is a heterogeneous disease with poor prognosis. Increasing evidence has revealed that microRNAs (miRNAs) are strongly associated with the pathogenesis and progression of MM. MiR‐637 promotes apoptosis and suppresses cell proliferation and autophagy in multiple myeloma by targeting NUPR1. This study provides a novel possible therapeutic target for MM.

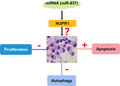

**Abbreviations**
BM: bone marrow
CCK‐8: cell counting Kit‐8
HDs: healthy donors
miR‐637: microRNA‐637
miRNAs: microRNAs
MM: multiple myeloma
NC: nonspecific control
NC‐LV: nonspecific control lentiviral vector
NUPR1: nuclear protein 1
NUPR1‐LV: NUPR1 lentiviral vector
OD: optical density

Multiple myeloma (MM) is a hematological malignancy characterized by the proliferation of malignant monoclonal plasma cells. It accounts for approximately 15% of all hematological malignancies [1]. However, existing treatment strategies are still not satisfactory [2]. Therefore, there is an urgent need to develop therapeutic targets based on the pathogenesis and progression of MM.

MicroRNAs (miRNAs) are a group of small, noncoding RNAs 18–25 nucleotides long that bind to the 3'‐untranslated region of their target mRNAs to regulate gene expression, causing target degradation or translation inhibition [3]. miRNAs are essential regulators of cell function, including cell proliferation, differentiation, and apoptosis [4]. Recently, several studies have shown that miR‐637 is clinically significant and has a crucial role in carcinogenesis and cancer progression of papillary thyroid carcinoma, pancreatic ductal adenocarcinoma, glioma, and hepatocellular carcinoma [5, 6, 7]. However, the role of miR‐637 in MM and the molecular mechanisms behind its role are still unknown.

NUPR1, also known as p8 and candidate of metastasis 1, has become a relevant target for the MM mechanism research. Our previous study showed that NUPR1 silencing significantly inhibited the proliferation of U266 and RPMI8226 cell lines and induced apoptosis *in vitro* [8]. NUPR1 has been proven to be the target of multiple miRNAs [9]. A previous study showed that miRNA‐325‐3p prevents sevoflurane‐induced learning and memory impairment by inhibiting NUPR1 [10]. So far, a miRNA that plays a specific role in MM progression has not been identified.

Bioinformatics analysis (TargetScan, miRDB, Starbase, and miRWalk) suggests that miR‐637 could target NUPR1. However, it remains unknown whether miR‐637 targets NUPR1 in an experiment. Hence, this study aimed to clarify the role of miR‐637 in MM and its therapeutic potential. Here, we report, for the first time, that miR‐637 is downregulated in the bone marrow of MM patients and MM cell lines. It is, therefore, widely proved that miR‐637 directly targets NUPR1 and further regulates proliferation, autophagy, and apoptosis in MM cells. Based on these results, we concluded that miR‐637 might be a promising new target for MM treatment.

## Materials and methods

### Clinical specimens

Bone marrow (BM) specimens were collected from 36 MM patients (18 males and 18 females; age range, 45–74 years) and 21 healthy donors (HDs) (10 males and 11 females; age range, 23–52 years) from the First Affiliated Hospital of Chongqing Medical University from September 2018 to November 2019. The patients’ details are listed in Table 1. Patients’ clinical information is provided as supplementary materials (see supplementary Table S1). Informed written consent was obtained from each MM patient and healthy donor, and the study protocol was approved by the Ethics Committee of Chongqing Medical University. The use of human samples complies with the standards stipulated in the Declaration of Helsinki. The Ethics Committee Approval is provided as Supporting Information. The study excluded patients undergoing radiotherapy, chemotherapy, immunoregulatory drugs, proteasome inhibitors, and autologous stem cell transplantation. Plasma cells from bone marrow were purified with anti‐CD138 magnetic‐activated cell separation microbeads (Miltenyi, Germany) according to the manufacturer's instructions, and the plasma cell purity was over 95%. The total RNA of the samples was extracted, reverse‐transcribed into cDNA, and stored at −80 °C until use.

**Table 1:** Correlations between miR‐637 expression in MM and clinical characteristics (*n* = 36)

Feature	No.	Expression of miR‐637 (mean ± SD)	*P* value
*Gender*			
Male	18	0.3583 ± 0.09758	0.4358
Female	18	0.3296 ± 0.1193	
*Age*			
<50	6	0.3353 ± 0.03941	0.834
≥50	30	0.3457 ± 0.1179	
*M component at diagnosis*			
IgG type	16	0.3537 ± 0.1019	0.0318
IgA type	11	0.3814 ± 0.09894	
IgD type	0		
IgM type	0		
Light chain type	9	0.2647 ± 0.09282	
*ISS stage*			
I	2		0.0035
II	4	0.4947 ± 0.0907	
III	30	0.3249 ± 0.1022	
*RISS stage*			
Stage I	1		0.0095
Stage II	4	0.4749 ± 0.09631	
Stage II	31	0.3278 ± 0.101	

### Primary samples and treatments

Primary neoplastic plasma cells were obtained at diagnosis from four patients of the First Affiliated Hospital of Chongqing Medical University. MM diagnosis was made in accordance with the International Myeloma Working Group criteria [11]. Informed consent was obtained from each patient in accordance with the guidelines of the local ethics policy. Mononuclear cells from bone marrow samples were isolated by Ficoll and directly cultured for 24 h in RPMI‐1640 (Thermo Fisher, Waltham, MA, USA) medium containing 10% fetal bovine serum (PAN, Bayern Munchen, Germany).

### Cell culture

MM cell lines (U266 and RPMI8226) were provided by Professor Jian Hou of Renji Hospital, Shanghai Jiaotong University School of Medicine (Shanghai, China). The cells were cultured in RPMI‐1640 media from Gibco (Thermo Fisher), supplemented with 10% fetal bovine serum (PAN), 100 μg·mL^−1^ streptomycin, and 100 U·mL^−1^ penicillin (Invitrogen) followed by incubation at 37 °C under 5% CO_2_. Given the growth status of cells, the culture medium was replaced per 2–3 days.

### Cell transfection

MM cells were seeded in a 6‐well plate at 5 × 10^5^ cells/well in a complete medium at 37 °C in 5% CO_2_. Subsequently, the cells were transfected with miR‐637 using Lipofectamine™ 3000 Transfection Reagent: miR‐637 mimics: (sense) 5′‐ACUGGGGGCUUUCGGGCUCUGCGU‐3′, (antisense) 5′‐GCAGAGCCCGAAAGCCCCCAGUUU‐3′; miR‐637 inhibitors: (sense) 5′‐ACGCAGAGCCC GAAAGCCCCCAGU‐3′; mimic NC: (sense) 5′‐UUCUCCGAACGUGUCACGUTT‐3′, (antisense) 5′‐ACGUGACACGUUCGGAGAATT‐3′; and inhibitor NC: (sense) 5′‐CAGUACUUUUGUGUAGUACAA‐3′. miR‐637 mimics, miR‐637 inhibitors, and negative controls were all originated from GenePharma. After transfection, the 6‐well plate was incubated for 48 h at 37 °C in 5% CO_2_ for subsequent experiments.

NUPR1 lentiviral vectors (NUPR1‐LV) and nonspecific control (NC‐LV) were synthesized as previously described by Shanghai GeneChem (Shanghai, China). U266 and RPMI8226 cell lines were transfected with LV, and puromycin was used to select cells with functional transfection status (Solarbio, Beijing, China). Cells expressing the green fluorescent protein were identified under a fluorescent microscope, and the transfection efficiency was evaluated by flow cytometry.

### Quantitative real‐time PCR

Total RNA was isolated from the cells using RNAiso Plus (Takara, Kusatsu, Japan) according to the manufacturer's instructions. The concentration and purity of RNA were measured with a spectrophotometer (NanoDrop 2000; Thermo Fisher). To harvest complementary DNA, 1 μg of RNA underwent reverse transcription with the PrimeScript™ RT Reagent Kit (Takara). The expression levels of miR‐637 and mRNAs were measured with TB Green^®^ Premix Ex Taq™ II (Takara) and a CFX96 Quantitative Real‐time PCR System (Applied Biosystems, Foster City, CA, USA). Reaction conditions included pre‐denaturation at 95 °C for 30 s, denaturation at 95 °C for 5 s, annealing at 60 °C for 30 s, 40 cycles, melt curve analysis. The relative expression levels of miR‐637 and mRNA were normalized to U6 or β‐actin using the 2^−△△Ct^ method (Livak and Schmittgen, 2001). The primer sequences used were as follows: miR‐637 (forward, 5′‐ACACTCCAGCTGGGACTGGGGGCTTTCGGGCT‐3′, and reverse, 5′‐CTCAACTGGTGTCGTGGAGTCGGCAATTCAGTTGAGACGCAGAG‐3′), U6 (forward, 5′‐CAGCACATATACTAAAATTGGAACG‐3′, and reverse, 5′‐ACGAATTTGCGTGTCATCC‐3′), NUPR1 (forward, 5′‐AGGACTTATTCCCGCTGACTGA‐3′, and reverse, 5′‐TGCCGTGCGTGTCTATTTATTG‐3′), and β‐actin (forward, 5'‐CCACGAAACTACCTTCAACTCC‐3', and reverse, 5'‐GTGATCTCCTTCTGCATCCTGT‐3').

### Target identification and dual‐luciferase assay

The miR‐637 target was identified by TargetScan 7.2 (http://www.targetscan.org/vert_72/), miRDB (http://mirdb.org/), Starbase(http://starbase.sysu.edu.cn/starbase2/index.php), and miRWalk (http://mirwalk.umm.uni‐heidelberg.de/). For the luciferase assay, the human NUPR1 3′‐UTR was substituted by the 3′‐UTR containing the complementing seed sequence of miR‐637 and synthesized into the pSI‐Check2 vector. The wild‐type (wt) NUPR1 3′‐UTR plasmid was also constructed into the pSI‐Check2 vector. MM cells were grown in 96‐well plates and cotransfected with miR‐637 mimics or nonspecific control (NC) and pSI‐Check2‐NUPR1‐mut‐3′‐UTR or wt pSI‐Check2‐NUPR1‐3′‐UTR with Transfection Reagent (Hanbio Biotechnology, Shanghai, China). A dual‐luciferase reporter system (Luciferase Assay Reagent; Promega, San Luis Obispo, California, USA) was used for luciferase activity detection.

### Cell Counting Kit‐8 assay

U266 and RPMI8226 cells were seeded into 96‐well plates at a concentration of 1 × 10^5^ cells/well. 90 uL of the medium and 10 uL of the Cell Counting Kit‐8 (CCK‐8) (Bimake, Houston，TX, USA) were added to each well. After incubating the plates for 2 h, the optical density (OD) values of the 1d, 2d, 3d, 4d, and 5d plates were measured with a microplate reader (Thermo Fisher Scientific) at an absorbance of 450 nm.

### Western blot analysis

We used a Total Protein Extraction Kit (Roche, Basel, Switzerland) to extract total protein from cells or samples and sodium dodecyl sulfate and polyacrylamide gel electrophoresis (SDS/PAGE) to separate proteins. Then, we transferred the protein to a PVDF membrane (Millipore, Darmstadt, Germany). Membranes were blocked with QuickBlock™ Blocking Buffer (Beyotime, Shanghai, China) for 15 min and washed with TBST three times. The membrane was incubated with the primary antibody at 4 °C overnight and then incubated with the secondary antibody at ambient temperature for 1 h. Following incubation with the secondary antibody, the membranes were washed with TBST. Subsequently, enhanced chemiluminescence reagents (Millipore) were used to detect the protein bands. The primary antibody for Bax, Bcl2, cleaved caspase 3, P62, LC3, and NUPR1 was provided by Abcam.

### Flow cytometry

MM cells were trypsinized and washed twice with cold PBS. According to the manufacturer's instructions, apoptotic cells were double‐stained with Annexin V‐APC/PI (Sungene, China). After incubation for 10–20 min in the dark, the cells were subjected to flow cytometry with a FACSCanto 6‐Color Flow Cytometer (BD Biosciences, San Jose, CA, USA).

### Statistical analysis

All experiments were conducted in triplicate, and the data are denoted as mean ± stand deviation (SD). prism 7.0 software (GraphPad; San Diego, CA, USA) was employed for statistical analysis. Comparison between two groups was evaluated using Student's *t*‐test. Multiple comparisons were assessed using one‐way analysis of variance (ANOVA) with LSD post hoc test. P‐values of *P < 0.05, **P < 0.01, and ***P < 0.001 were considered to exhibit statistical significance.

## Results

### miR‐637 was downregulated in the MM samples

**Fig. 1:**
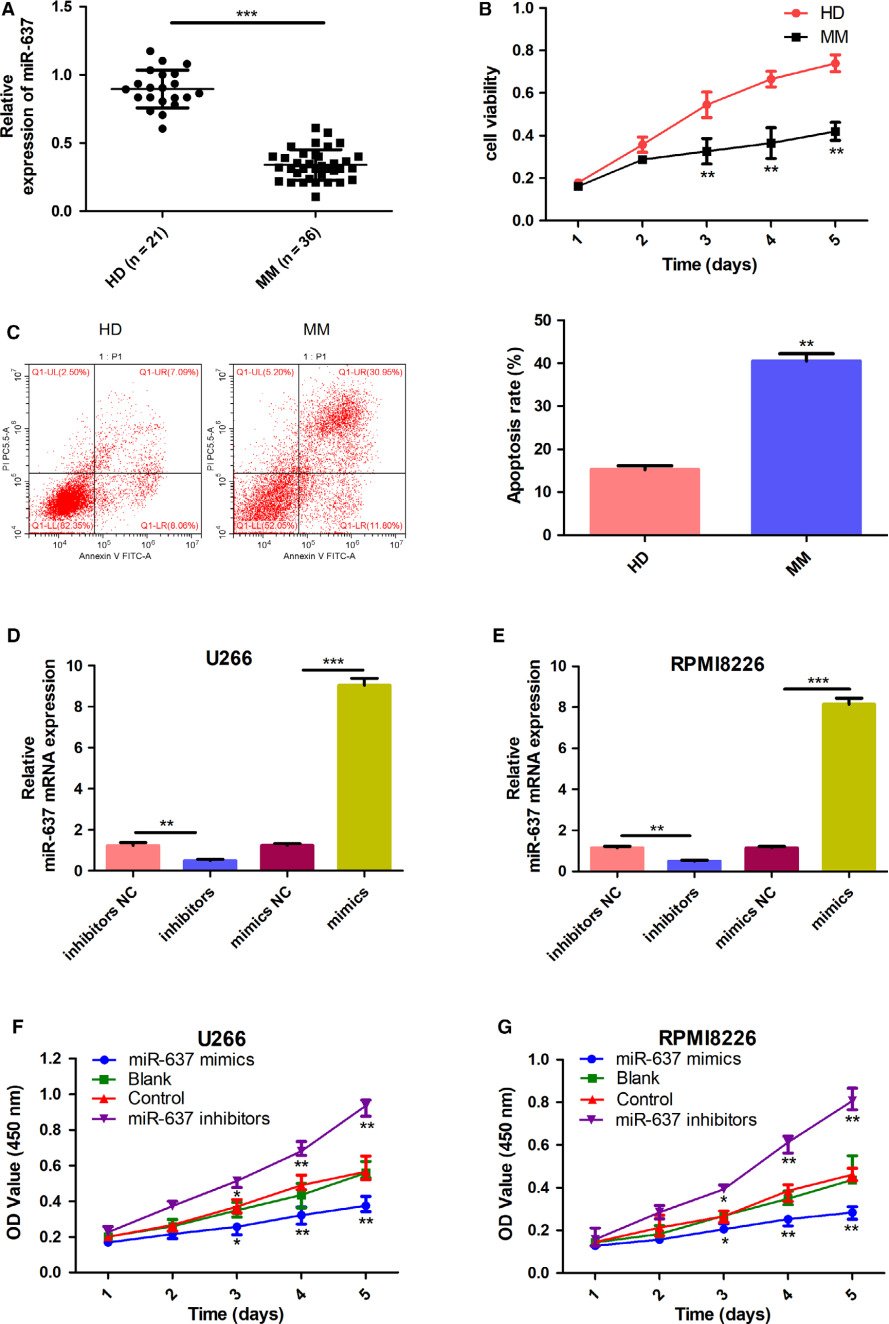
Low expression of miR‐637 is involved in the viability and apoptosis of primary MM cells and MM cell lines. (A) The relative expression of miR‐637 was measured by RT–PCR both in MM samples (*n* = 36) and in healthy donors (HD) samples (*n* = 21). (B) The viability of the primary HD and MM cells was detected by CCK‐8 assay. (C) The apoptotic cells of HD and MM were evaluated by flow cytometry (*n* = 3, Student’s *t*‐test). (D, E) The relative expression of miR‐637 in U266 and RPMI8266 cells was measured by RT–PCR in the inhibitor NC, miR‐637 inhibitors, mimic NC, and miR‐637 mimic groups (*n* = 3, Student’s *t*‐test). (F, G) The cell viability of U266 and RPMI8266 in each group was determined by the CCK‐8 assay; comparison was performed between the miR‐637 mimic/inhibitor group and the control group. miR‐637 mimics significantly decreased the viability rate both in U266 cells and in RPMI8266 cells, as compared with the vector control group at 1d, 2d, 3d, 4d, and 5d (*P* = 0.031, 0.007, and 0.003 in U266 cells, respectively; *P* = 0.034, 0.002, and 0.008 in RPMI8266 cells, respectively). Transfection of miR‐637 inhibitors significantly increased the viability rate both in U266 cells and in RPMI8266 cells, as compared with its vector control at 1d, 2d, 3d, 4d, and 5d (*P* = 0.048, 0.009, and 0.004 in U266 cells, respectively; *P* = 0.028, 0.003, and 0.009 in RPMI8266 cells, respectively). All data are shown as mean ± SD of three independent experiments. *P < 0.05, **P < 0.01, and ***P < 0.001.

RT–PCR was applied to evaluate the expression levels of miR‐637 in the samples of MM patients and healthy donor samples. The results showed that the average expression level of miR‐637 in the MM samples was significantly lower than that in control samples (*P* < 0.01, Fig. 1A), indicating an abnormal downregulation of miR‐637 in the bone marrow of MM patients. Thus, miR‐637 might play an inhibitory role in the development of MM.

### miR‐637 suppressed proliferation and promoted apoptosis in MM primary cells

The cell viability of the primary cells from HD and MM patients was evaluated by CCK‐8 assay (Fig. 1B). Annexin V/PI staining of the HD‐ and MM‐transfected primary cells was performed to determine whether miR‐637 induced apoptosis in MM primary cells (Fig. 1C). The results showed that miR‐637 overexpression caused induction of apoptotic cell death of MM primary cells. Collectively, we demonstrated that miR‐637 mimics inhibited growth and induced apoptosis in MM primary cells.

### miR‐637 suppressed proliferation and autophagy and promoted apoptosis in MM cells

**Fig. 2:**
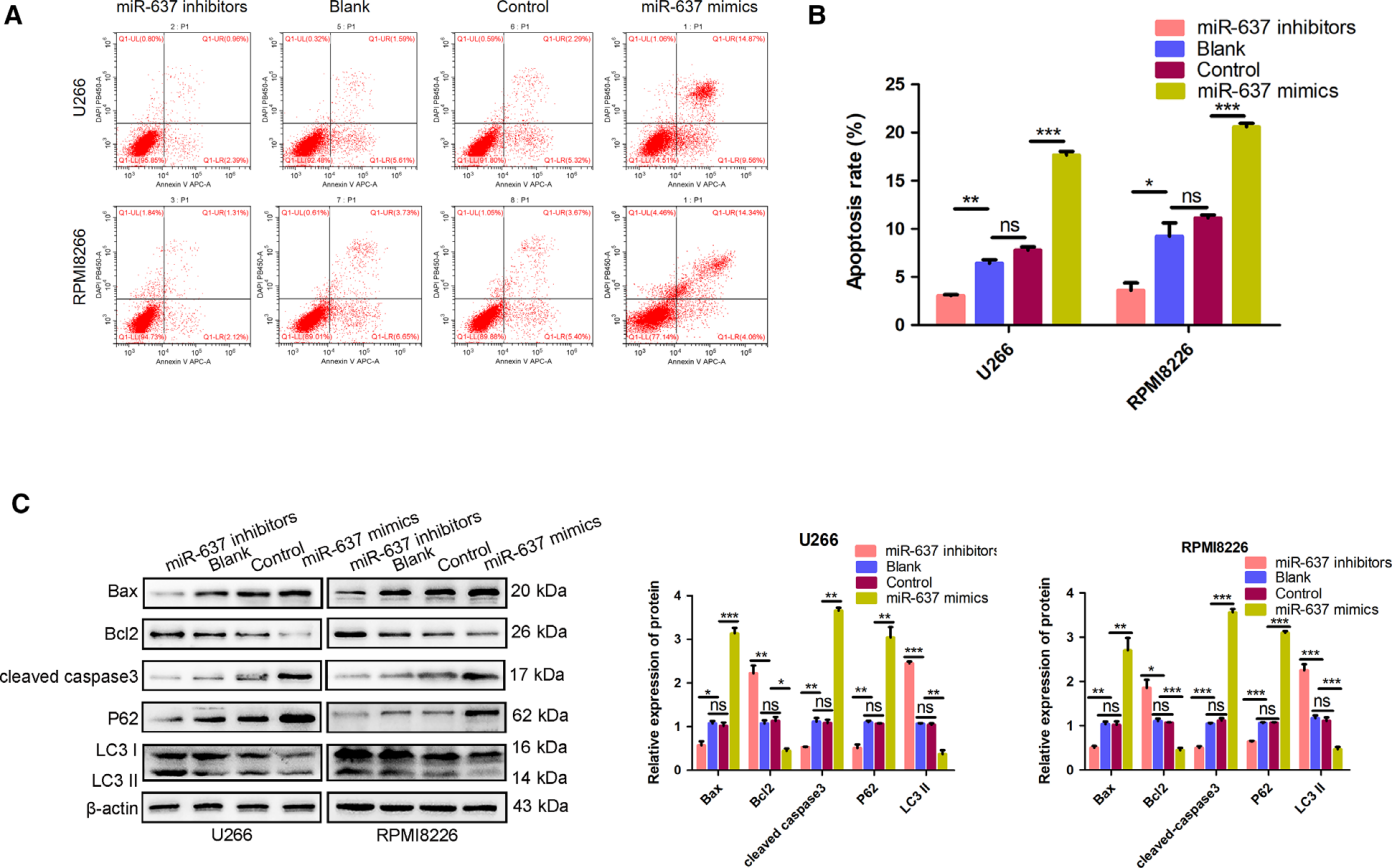
miR‐637 induced cell apoptosis and suppressed autophagy activities in U266 and RPMI8226 cells. (A, B) Annexin V/PI staining of the control, negative control, miR‐637 mimics, and miR‐637 inhibitor groups of U266 and RPMI8226 cells was performed by flow cytometry. Apoptotic rates in MM cells were calculated (*n* = 3, Student’s *t*‐test). (C) The protein expression of Bax, Bcl2, cleaved caspase 3, LC3, and P62 in each group was detected by western blot. β‐actin was used for loading control. All data are shown as mean ± SD of three independent experiments (*n* = 3, one‐way ANOVA). *P < 0.05, **P < 0.01, and ***P < 0.001.

To reveal the effect of miR‐637 on cell proliferation, mimic NC, miR‐637 mimics, inhibitor NC, and miR‐637 inhibitors were transfected into MM cells. RT–PCR confirmed the overexpression of miR‐637 in the mimic groups and the downregulation in the inhibitor groups (Fig. 1D, E). After that, CCK‐8 assay was used to monitor the cell viability at different time intervals. The results showed that the transfection of MM cells with miR‐637 mimics could cause a significant decrease in the cell viability, and miR‐637 inhibitors presented higher cell viability compared with the NC (Fig. 1F, G). We also treated the transfected cells of NC, miR‐637 mimics, and miR‐637 inhibitors with Annexin V/PI staining to study the role of miR‐637 in cell apoptosis. The results indicated that for the miR‐637 mimic‐transfected U266 and RPMI8226 cells, the apoptotic rates increased significantly (Fig. 2A,B). Moreover, western blot showed the upregulation of Bax and cleaved caspase‐3 lysis, and the downregulation of Bcl2, in the mimic groups, which were in contrast with those in the inhibitor groups. Further evidence of autophagy triggered by miR‐637 was demonstrated by evaluating the expression of p62 and LC3 in MM cells. As expected, miR‐637 mimics caused a lower ratio of LC3 and higher expression levels of p62 than the blank and control groups, with miR‐637 inhibitors showing the opposite (Fig. 2C). In total, the above findings indicated that miR‐637 inhibited proliferation and autophagy, and induced apoptosis in MM cells.

### miR‐637 targeted NUPR1 in MM cells

**Fig. 3:**
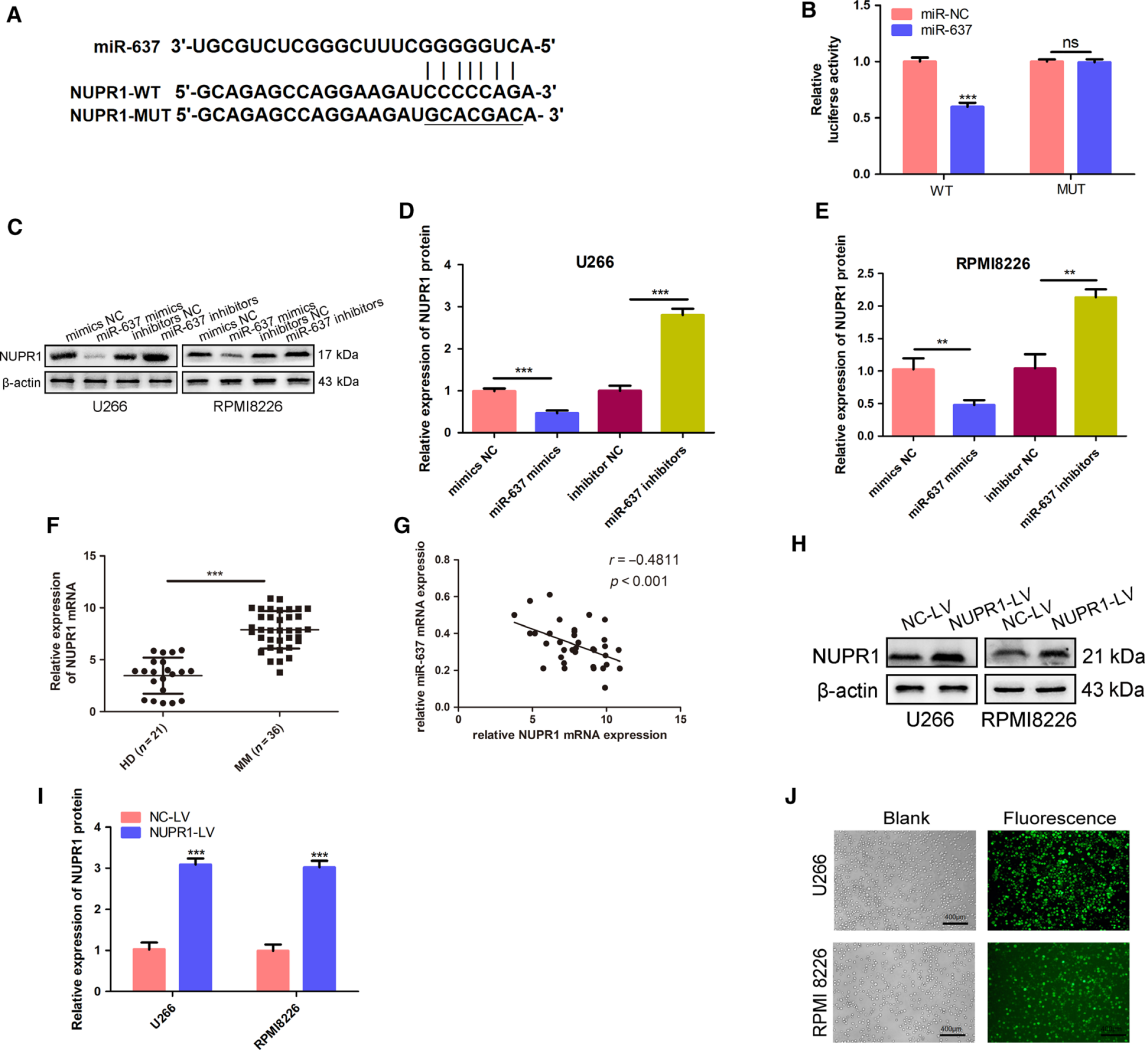
miR‐637 targeted NUPR1 in MM cells. (A) Data of bioinformatics tools (TargetScan) showed that miR‐637 was able to bind to the 3’‐UTR of NUPR1. (B) Luciferase activity was measured in MM cells (*n* = 3, Student’s *t*‐test). (C–E) The protein expression of NUPR1 in the MM cells was detected by western blot in the mimic NC, miR‐637 mimics, inhibitor NC, and miR‐637 inhibitor groups. β‐actin was used for loading control (*n* = 3, Student’s *t*‐test). (F) The relative mRNA expression of NUPR1 in the bone marrow samples of MM patients (*n* = 36) and healthy donors (*n* = 21) was measured using RT–PCR. (G) The negative association was observed between the expressions of miR‐637 and NUPR1 using Pearson's correlation coefficient analysis (*P* < 0.001, *r* = −0.4881). (H and I) The protein expression of NUPR1 in the NC‐LV and NUPR1‐LV groups was determined by western blot. β‐actin was used for loading control (*n* = 3, Student’s *t*‐test). (J) The expression of green fluorescent protein presented the transfection efficiency of NC‐LV and NUPR1‐LV under a fluorescent. Scale bars: 400 μm. All data are shown as mean ± SD of three independent experiments. *P < 0.05, **P < 0.01, and ***P < 0.001.

To further investigate the mechanism of miR‐637, we conducted TargetScan bioinformatics analyses. The results showed that NUPR1 was a potential target of miR‐637 (Fig. 3A), which was verified by a dual‐luciferase assay (Fig. 3B). The transfection with the miR‐637 mimics significantly reduced the mRNA and protein expression levels of NUPR1. In contrast, transfection with miR‐637 inhibitors increased NUPR1 expression levels compared with NCs (Fig. 3C‐E). Furthermore, we found that NUPR1 mRNA expression level in the bone marrow samples of MM patients (*n* = 36) was significantly higher compared with that in the healthy individuals (*n* = 21) by RT–PCR (Fig. 3F). Moreover, NUPR1 expression was negatively correlated with miR‐637 in the patients’ samples (Fig. 3G). The aforementioned results suggested that miR‐637 might play roles by targeting NUPR1 in MM cells.

### NUPR1 mediated the effect of miR‐637 on MM cells

**Fig. 4:**
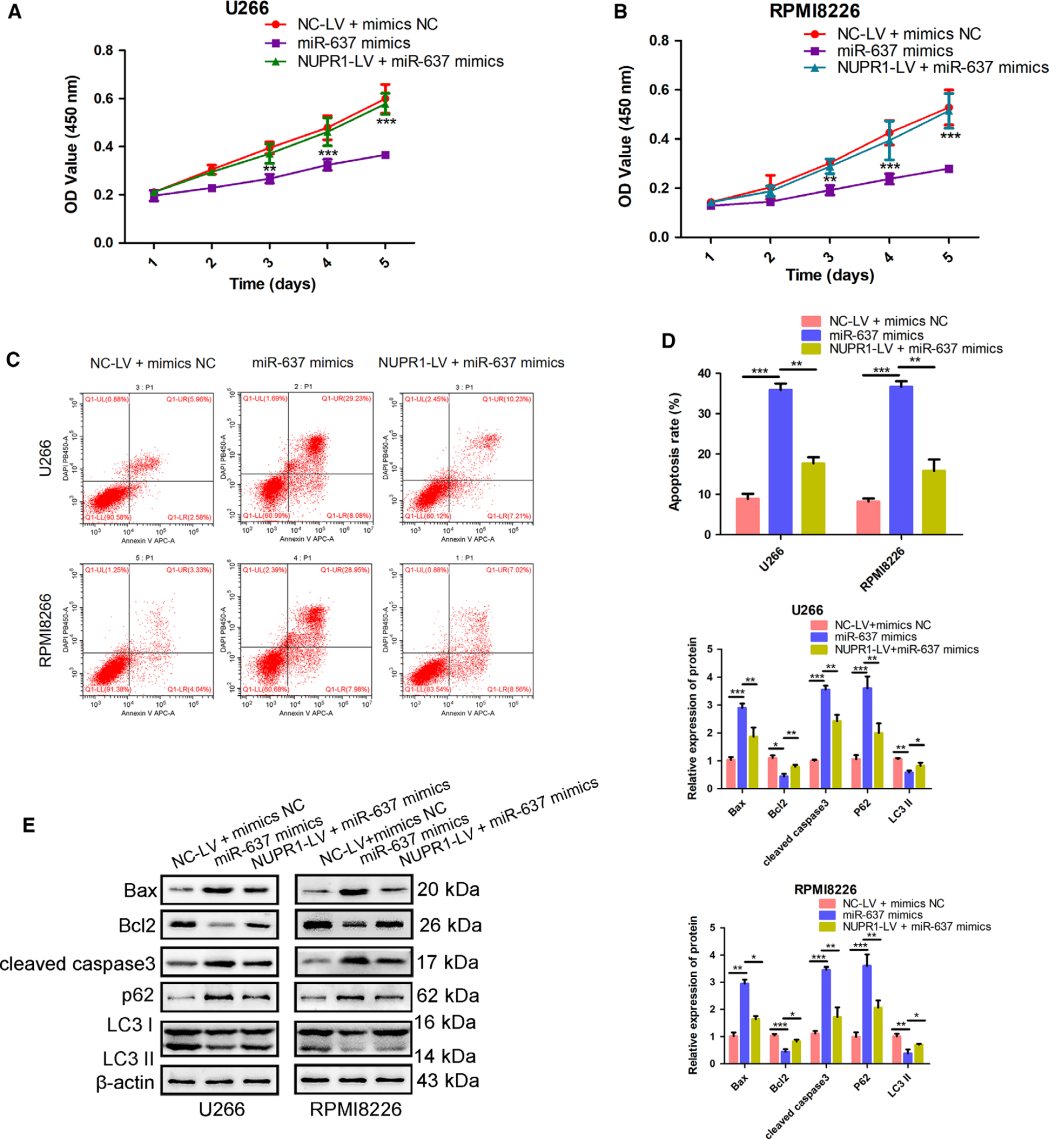
Overexpression of NUPR1 partially reversed the role of miR‐637 in inhibiting proliferation, autophagy, and promoting apoptosis. (A, B) The cell viability of MM cells after transfecting miR‐637 mimics, NC‐LV, or NUPR1‐LV was determined by CCK‐8 assay. Comparison was performed between the NUPR1‐LV + miR‐637 mimic group and the miR‐637 mimic group. The viability rate of U266 cells was higher in the NUPR1‐LV + miR‐637 mimic group than that in the miR‐637 mimic group at 1d, 2d, 3d, 4d, and 5d (*P* = 0.042, 0.005, and 0.003, respectively). Likewise, the viability rate of RPMI8266 in the NUPR1‐LV + miR‐637 mimic group was significantly increased, as compared with miR‐637 mimics at 1d, 2d, 3d, 4d, and 5d (*P* = 0.049, 0.006, and 0.007, respectively). (C, D) The apoptosis of MM cells was detected by flow cytometry (*n* = 3, Student’s *t*‐test). (E) The protein expression of Bax, Bcl2, cleaved caspase 3, P62, and LC3 was detected by western blot. β‐actin was served as loading control. Data are shown as mean ± SD of three independent experiments (*n* = 3, one‐way ANOVA). *P < 0.05, **P < 0.01, and ***P < 0.001.

To verify whether NUPR1 mediated the effect of miR‐637 on MM cells, including suppressing proliferation and autophagy, and promoting apoptosis, we transfected NUPR1‐LV to U266 and RPMI8226. The expressing green fluorescent protein showed transfecting efficiency (Fig. 3J). NUPR1 overexpression was also verified by western blot (Fig. 3H, I). As shown in Fig. 4A,B, NUPR1 overexpression reversed the inhibiting effect of miR‐637 on cell proliferation. In addition, NUPR1 overexpression could inhibit miR‐637 mimic‐induced apoptosis. Western blot analysis also showed that NUPR1 overexpression reversed the apoptosis‐inducing effect (Fig. 4C, D) and autophagy‐inhibiting effect (Fig. 4E) of miR‐637 mimics. To summarize, NUPR1 was directly involved in the regulation of cell proliferation, autophagy, and apoptosis of miR‐637 on MM cells.

## Discussion

Multiple myeloma is one of the most common hematological malignancies [12]. Studies have shown that multiple miRNAs act as regulators in the progression of MM by participating in cell proliferation, apoptosis, and metastasis [13]. Many researchers have mentioned the potential of miRNAs as therapeutic targets in MM [14]. Some results revealed that miR‐637 hindered the development of melanoma, colorectal cancer, cervical cancer, and gastric cancer [15, 16, 17]. Moreover, miR‐637 has been suggested as a tumor suppressor in oral squamous cell carcinoma [18]. In the present study, the overexpression of miR‐637 in MM cells caused a significant reduction in proliferation and autophagy but a promotion in apoptosis, showing a tumor‐suppressive role in MM. The result is in accordance with the previous report exhibiting that the overexpression of miR‐637 inhibited proliferation and induced apoptosis [17].

In this study, the expression level of miR‐637 was first explored in 36 human samples, and it was found that the expression of miR‐637 in the bone marrow samples of MM patients was significantly lower than that of control healthy samples. Since miR‐637 was downregulated in MM samples, we speculated that miR‐637 played a carcinogenic role in MM. According to this speculation, we further demonstrated that the expression of miR‐637 could suppress cell proliferation and autophagy activity, and promote apoptosis in MM, indicating that miR‐637 takes part in the development of MM.

To clarify the mechanism of miRNA in MM, it is required to determine the targeted gene regulated by the miRNA. It is reported that miR‐215‐5p is an anti‐oncogene in MM‐targeting RUNX1 [19]. The analyses of bioinformatics databases showed NUPR1 as the main target of miR‐637 in MM cells. However, there may be some uncertainty in bioinformatics methods, which requires further research and conformation by experiments.

NUPR1 was evaluated as a possible candidate. NUPR1 is an important multifunctional protein involved in cell stress response, regeneration and cell growth, and carcinogenesis, including MM [8]. NUPR1 is upregulated in the bone marrow of patients with MM and is highly correlated with the clinical features of MM [20]. Downregulation of NUPR1 could significantly inhibit cell proliferation and promote autophagy‐mediated apoptosis in MM. In our study, a luciferase reporter gene assay was performed to determine whether miR‐637 could bind to the 3'‐UTR of NUPR1, making NUPR1 a direct target of miR‐637. We further verified the overexpression of NUPR1 and concluded that miR‐637 expression was negatively correlated with NUPR1 in the samples of MM patients. *In vitro*, miR‐637 mimics inhibited the expression of NUPR1, while miR‐637 inhibitors increased the expression of NUPR1. The effect of miR‐637 overexpression is consistent with the role of knocking‐down NUPR1 in MM cells, that is inhibiting cell proliferation and autophagy, and promoting apoptosis. Both the miR‐637 mimics and the NUPR1‐LV were transfected into MM cells, and the results indicated that NUPR1 overexpression reversed the effect of miR‐637 on cell proliferation and autophagy, and apoptosis. It is, therefore, widely proved that miR‐637 directly targets NUPR1 and further regulates proliferation, autophagy, and apoptosis in MM.

## Conclusion

Overall, this study demonstrates the downregulation of miR‐637 in the bone marrow of MM patients and MM cell lines. *In vitro*, miR‐637 overexpression can suppress cell proliferation and autophagy and promote apoptosis through the direct target of NUPR1 in MM. Therefore, miR‐637 could be used as an anti‐oncogene and may provide a valid target for subsequent MM treatment.

## Conflict of interest

The authors declare no conflict of interest.

## Author contributions

X.C. designed the research, performed research, and wrote the manuscript; A.L. designed the study and performed experiments in vitro; Q.Z. collected clinical specimens and conducted specimen experiments; Z.J. provided technical support and analyzed clinical data; Y.C. collected data statistics, proofread, and revised the article; J.C. designed the research, provided financial support, and contributed to the writing and revisions. All authors approved the final version and submission of the manuscript.

## Supporting information


**Fig S1.** Low expression of miR‐637.

**Fig S2.** MiR‐637 regulates apoptosis and autophagy.
**Fig S3.** MiR‐637 targeted NUPR1.
**Fig S4.** Overexpression of NUPR1 reversed the role of miR‐637.

**Data S1.** Patient clinical information.

**Data S2.** Ethics approval.

## Data Availability

All data generated or analyzed during this study are included in this article.
