# Supplementary figures and images for: microRNA‐637 promotes apoptosis and suppresses proliferation and autophagy in multiple myeloma cell lines via NUPR1
Source: FEBS Open Bio. 2020 Dec 30;11(2):519–28. doi: 10.1002/2211-5463.13063 (PMC7876500; doi:10.1002/2211-5463.13063)

Blank Fluorescence


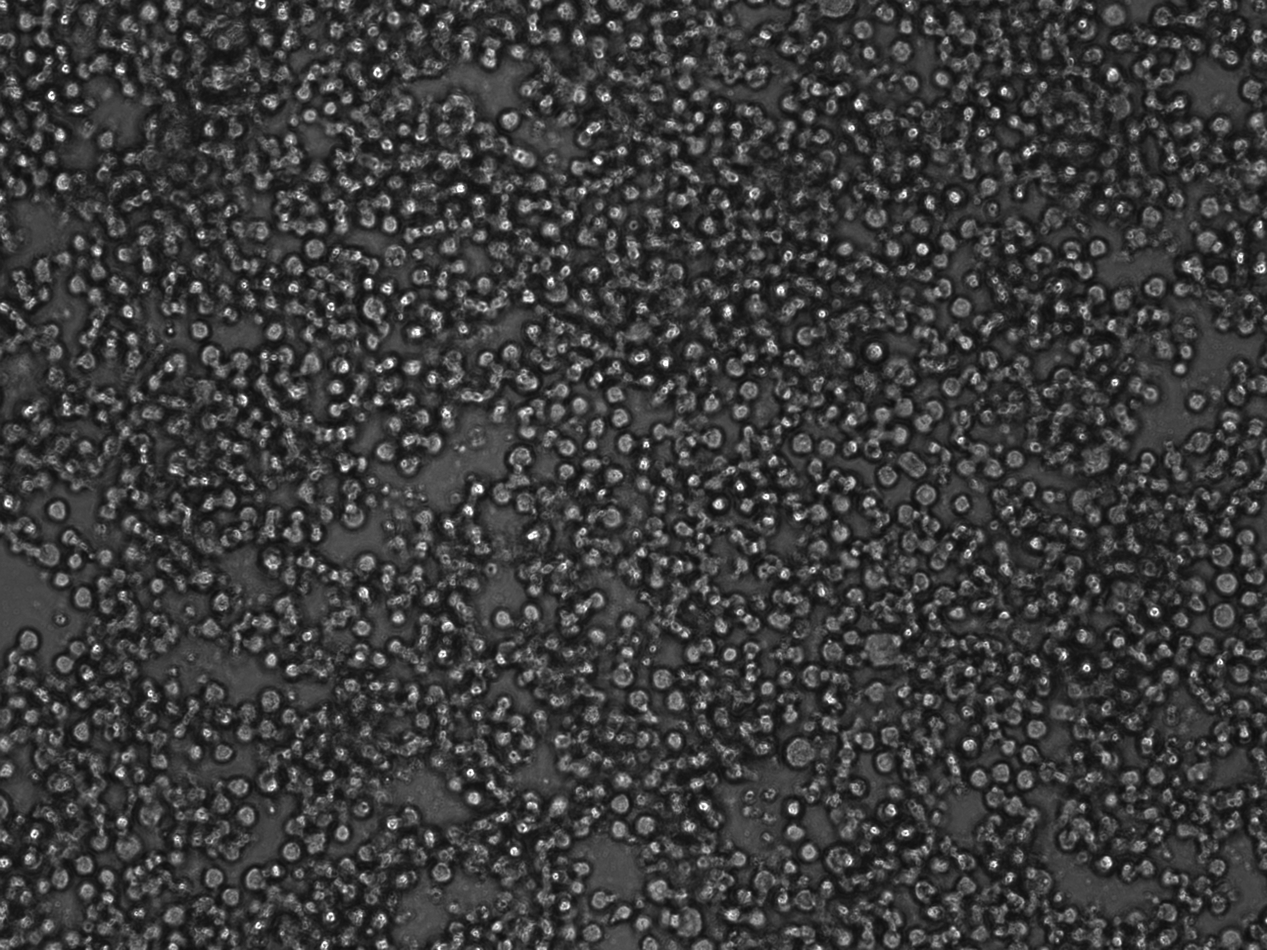

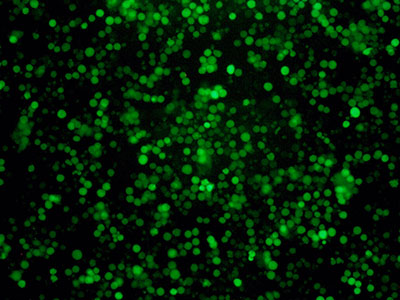

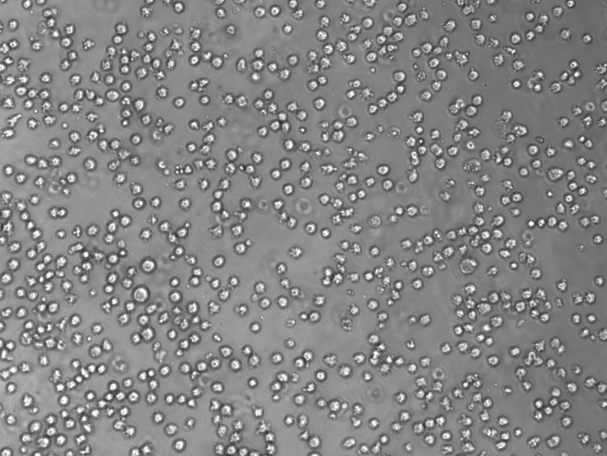

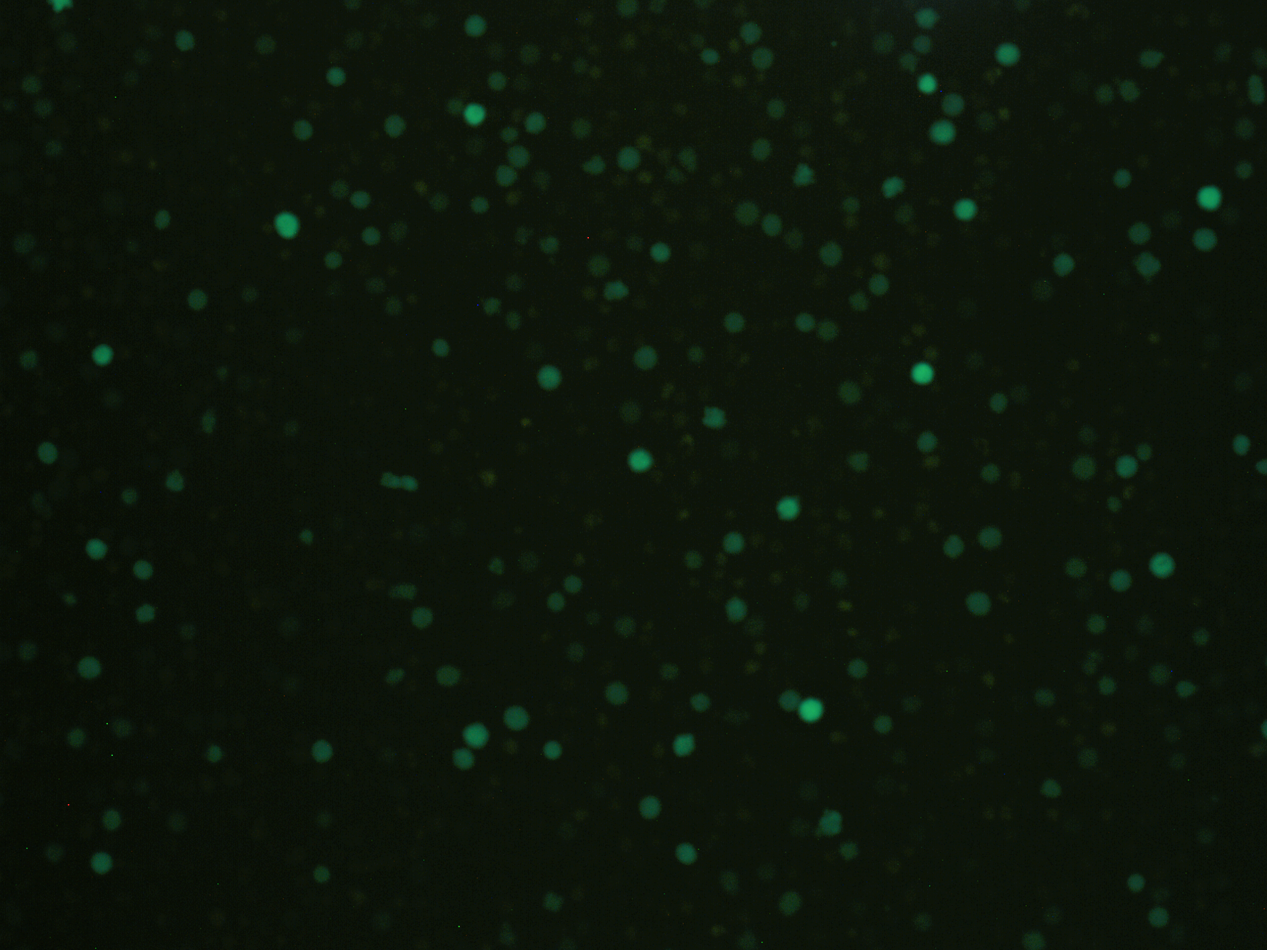

Supplement: Supplementary file 1 — Fig S1. Low expression of miR‐637. [file FEB4-11-519-s001.docx]

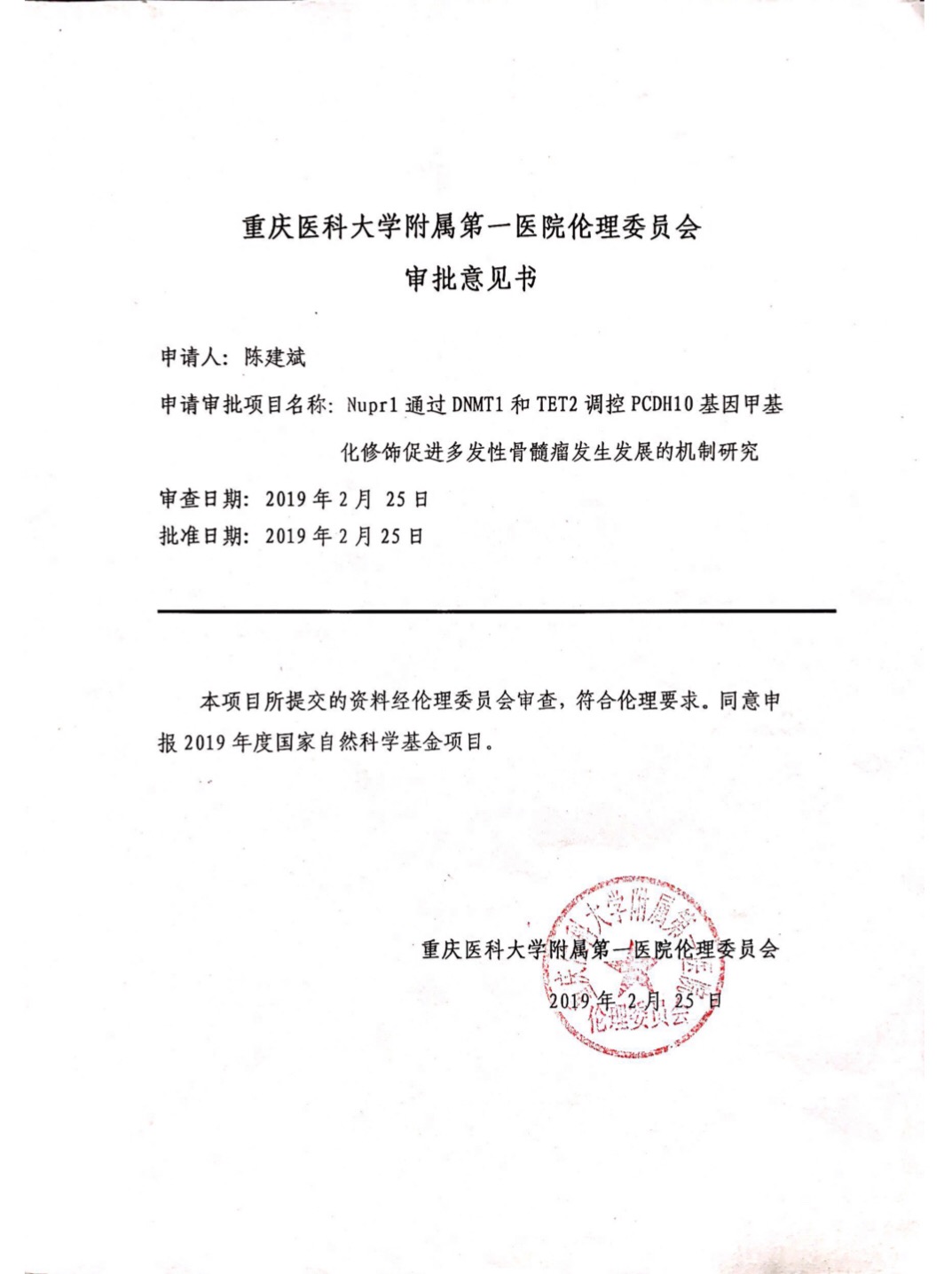

Supplement: Supplementary file 4 — Data S2. Ethics approval. [file FEB4-11-519-s004.doc]
